# Hypoxia-induced reprogramming of glucose-dependent metabolic pathways maintains the stemness of human bone marrow-derived endothelial progenitor cells

**DOI:** 10.1038/s41598-023-36007-5

**Published:** 2023-05-31

**Authors:** Dongni Lin, Kaihao Yan, Lingyun Chen, Junxiong Chen, Jianing Xu, Zijing Xie, Zhujun Li, Shuo Lin, Jinghuan Li, Zhenzhou Chen

**Affiliations:** 1 The Engineering Technology Research Center of Education Ministry of China, Guangdong Provincial Key Laboratory On Brain Function Repair and Regeneration, Department of Neurosurgery, The National Key Clinical Specialty, Zhujiang Hospital, Southern Medical University, 253# Gongye RD, Guangzhou, 510282 China; 2 Hygiene Detection Center, School of Public Health and Tropical Medicine, Southern Medical University, Guangzhou, 510515 China; 3 The Second School of Clinical Medicine, Undergraduate Innovation and Entrepreneurship Project, Southern Medical University, 253 Gongye Road, Guangzhou, 510282 China

**Keywords:** Cell growth, Metabolomics, Self-renewal

## Abstract

The benefits of hypoxia for maintaining the stemness of cultured human bone marrow-derived endothelial progenitor cells (BM EPCs) have previously been demonstrated but the mechanisms responsible remain unclear. Growing evidences suggest that cellular metabolism plays an important role in regulating stem cell fate and self-renewal. Here we aimed to detect the changes of glucose metabolism and to explore its role on maintaining the stemness of BM EPCs under hypoxia. We identified the metabolic status of BM EPCs by using extracellular flux analysis, LC–MS/MS, and ^13^C tracing HPLC-QE-MS, and found that hypoxia induced glucose metabolic reprogramming, which manifested as increased glycolysis and pentose phosphate pathway (PPP), decreased tricarboxylic acid (TCA) and mitochondrial respiration. We further pharmacologically altered the metabolic status of cells by employing various of inhibitors of key enzymes of glycolysis, PPP, TCA cycle and mitochondria electron transport chain (ETC). We found that inhibiting glycolysis or PPP impaired cell proliferation either under normoxia or hypoxia. On the contrary, inhibiting pyruvate oxidation, TCA or ETC promoted cell proliferation under normoxia mimicking hypoxic conditions. Moreover, promoting pyruvate oxidation reverses the maintenance effect of hypoxia on cell stemness. Taken together, our data suggest that hypoxia induced glucose metabolic reprogramming maintains the stemness of BM EPCs, and artificial manipulation of cell metabolism can be an effective way for regulating the stemness of BM EPCs, thereby improving the efficiency of cell expansion in vitro.

## Introduction

Bone marrow (BM)-derived endothelial progenitor cells (EPCs) participate in the repair of the vascular endothelium and in the neovascularization of ischemic tissue^1^. The potential of transplanted BM EPCs for treatment of diverse ischemic diseases has been demonstrated by animal experiments and by preliminary clinical trials^2,3^. However, low BM EPCs numbers in both peripheral blood and BM mean that cell expansion would be necessary for any clinical application. The maintenance of stemness thus becomes crucial both for improving expansion efficiency and for preserving the therapeutic effect. BM EPCs cultured under normoxia (~ 20% O_2_) showed reduced proliferation, colony formation, in vitro angiogenesis and contained a higher proportion of senescent cells compared with those cultured under hypoxia (1% O_2_)^4^. These findings suggest that hypoxia is more favorable for maintaining BM EPCs stemness than the normoxia of usual culture conditions. However, any mechanisms involved in maintenance of stemness by hypoxic conditions remain unclear.

Bioenergetic metabolism is fundamental to cell function providing a continuous, yet adaptable, energy supply. Emphasis has recently shifted from the study of cellular metabolism for energy production to its role in regulating stem cell fate and self-renewal^5^. The flexibility in flux through metabolic pathways accommodates a balance between anabolic processes to support biosynthesis (of nucleotides, phospholipids and amino acids) and catabolic processes to ensure adequate bioenergetic resources. Furthermore, metabolites have been shown to influence the epigenome through post-translational modifications of histones, DNA and transcription factors in both stem and differentiated cells. Therefore, there is an increasing awareness that cellular metabolism is not a passive player in stem cell lineage commitment but, rather, a determinant of stem cell fate. The role of metabolism in regulating cell fate has been termed “metabolic reprogramming”^6^.

Metabolism can be broadly divided into oxidative (mitochondrial) or non-oxidative (cytosolic). A switch from glycolysis to mitochondrial oxidation has been observed to accompany differentiation whereas the reverse switch from mitochondrial oxidation to glycolysis accompanies the reprogramming of mature somatic cells into induced pluripotential stem cells^5,7,8^. Hypoxic conditions have been shown to reprogram the metabolic mode of tumor cells, upregulating key glycolytic genes, such as glucose transporter 1, hexokinase 2 and lactate dehydrogenase A, to increase flux through glycolysis and decrease oxidative phosphorylation^9–11^. Such changes were accompanied by increased rates of conversion of pyruvate to lactate, rather than to acetyl CoA, and by decreased tricarboxylic acid (TCA) flux and nicotinamide adenine dinucleotide (NADH) delivery to the electron transport chain (ETC). Thus, hypoxia appears to be associated with cellular metabolic reprogramming and stemness. However, such roles of hypoxia in BM EPCs have not been systematically studied.

The current study examined changes in glucose metabolism and effects on BM EPCs stemness under hypoxia. The aim was to improve our knowledge regarding BM EPCs, increase expansion efficiency and thereby advance the use of BM EPCs transplantation in clinical settings.

## Results

### Cell stemness was maintained and metabolic reprogramming induced by hypoxia

**Figure 1:**
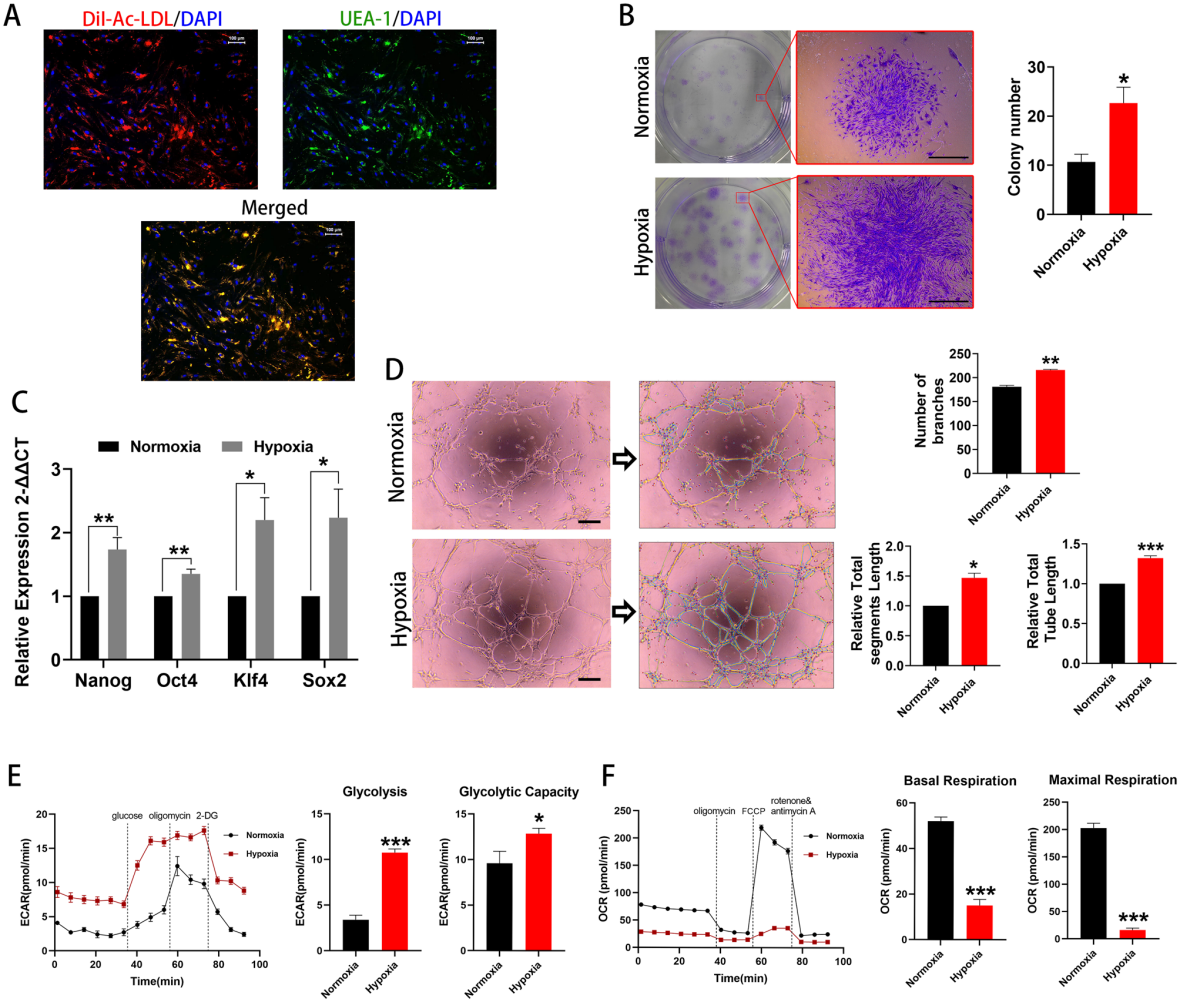
Cell stemness and kinetic metabolic profiling assessments. (**A**) Typical BM EPCs under normoxia were characterized by double positive staining (merged in yellow) with both DiI-AcLDL (red) and FITC-UEA-I (green). Scale bar represents 100 μm. (**B**) Representative images of BM EPCs colonies. Scale bar represents 1 mm. The number of BM EPCs colonies under hypoxia was significantly higher than that under normoxia (n = 6). (**C**) qRT-PCR results showing higher expression of the stemness markers, Nanog, Oct4, Klf4 and Sox2 under hypoxia than under normoxia (n = 5). (**D**) Representative tube networks formed by BM EPCs under normoxia and hypoxia, respectively (n = 3). Images analyzed by the ImageJ plugin “Angiogenesis analyze.” There is an indication of master junctions (pink dots), master segments (yellow), meshes (light blue), branches (green), and isolated segments (blue). Longer total segments length, longer total tube length, and more number of branches were found under hypoxia compared with normoxia. Scale bar represents 200 μm. (**E**) Representative experiment showing extracellular acidification rate (ECAR) of BM EPCs cultured under normoxia or hypoxia. ECAR representing higher glycolytic rate and capacity in BM EPCs under hypoxia than under normoxia (n = 6). (**F**) Representative experiment showing oxygen consumption rate (OCR) of BM EPCs cultured under normoxia or hypoxia. OCR for basal and maximal respiration in BM EPCs under hypoxia was lower than under normoxia (n = 6). Data are presented as Mean ± SEM. *p < 0.05; **p < 0.01; ***p < 0.001 versus BM EPCs under normoxia.

The human BM EPCs were previously shown to express HLA-ABC, CD105, CD90, CD44, and CD29; moderately expressed HLA-DR, KDR, and CD54; and negatively express CD34, CD31, CD45, or CD144^3^. These cells were also capable of DiI-Ac-LDL uptake and UEA-1 binding, consistent with endothelial lineage cells. (Fig. 1A). Figure 1B shows the colony forming ability of human BM-derived EPCs under normoxia (~ 20% O_2_) and hypoxia (1% O_2_). Colony formation increased to 228 ± 41% of the normoxic rate when hypoxic conditions were employed (p < 0.05). Expression of the stemness markers, Nanog, Oct4, Klf4 and Sox2, was higher under hypoxia than under normoxia (Fig. 1C), indicating more favorable maintenance of stem-like characteristics. To evaluate the functional characteristics of BM EPCs, compared with normoxia, the abilities of in vitro angiogenesis were better maintained under hypoxia, including longer total segments length, longer total tube length, and more number of branches (both p < 0.05, Fig. 1D).

A Seahorse Real-time Extracellular XFe96 Flux Analyzer^12^ was used to assess glycolytic function and mitochondrial respiration. Rates of glycolysis were increased to 366 ± 74% (p < 0.001) and maximal glycolytic capacity to 145 ± 19% (p < 0.05) under hypoxic compared with normoxic conditions (Fig. 1E). Rates of basal respiration decreased to about 30 ± 6% and of maximal respiration to 8 ± 2% (both p < 0.001) compared with normoxic conditions (Fig. 1F).

### Metabolite levels

**Figure 2:**
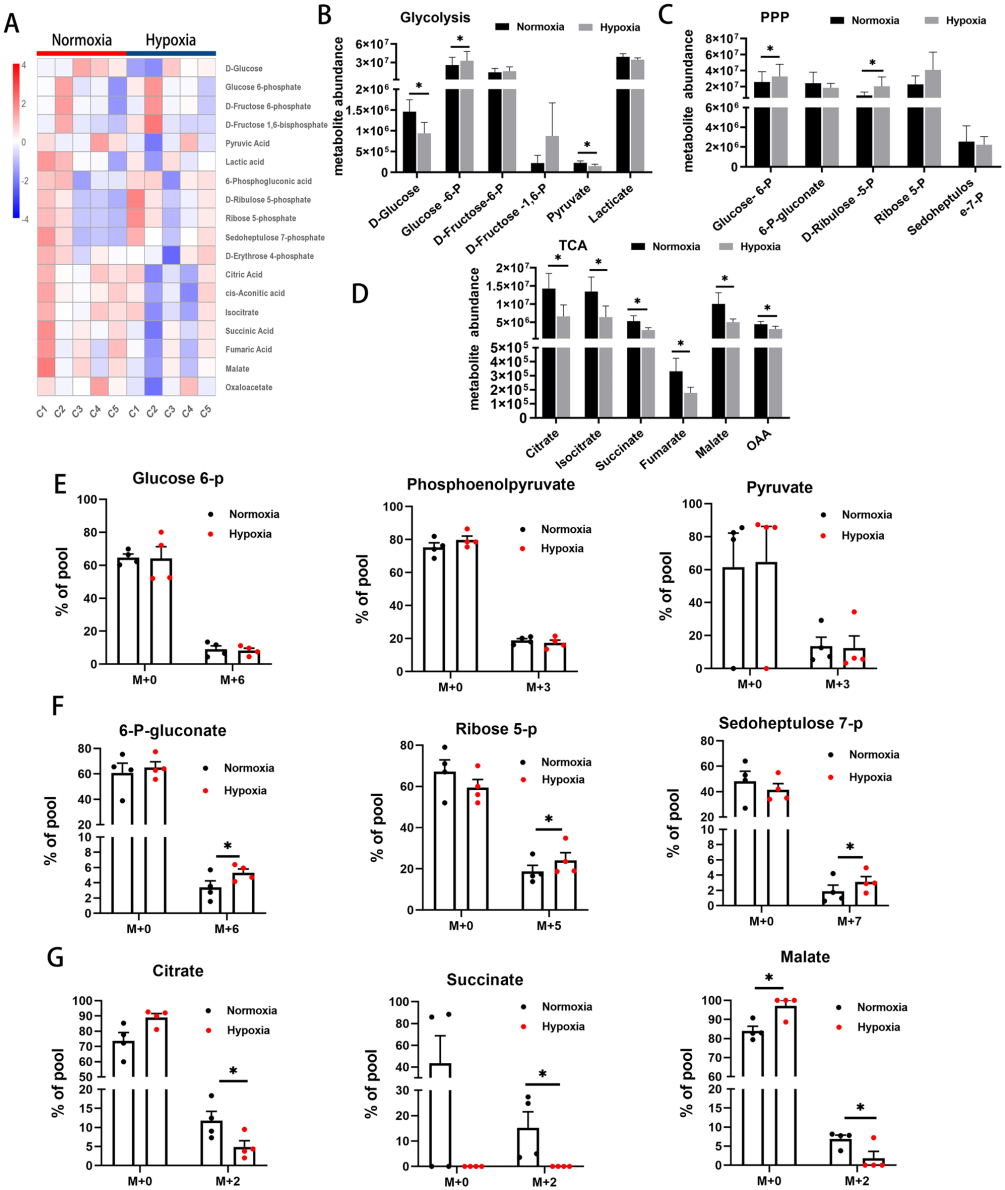
Glucose metabolic profiles and ^13^C tracing of intermediates of glucose metabolism. (**A**) Heatmap of intermediates of glucose metabolism detected by LC–MS (C1 ~ C5: case 1 ~ case 5). (**B–D**) Targeted analysis of abundance of different metabolites in major glucose-dependent metabolic pathways: glycolysis (**B**), PPP (**C**) and TCA cycle (**D**) using LC–MS (n = 5). (**E–G**) HPLC-QE-MS detection of ^13^C tracing carbon flow into intermediates of glycolysis (**E**), PPP (**F**) and TCA cycle (**G**). M + represents the number of carbons labeled with ^13^C (n = 4). Data are presented as Mean ± SEM. *p < 0.05 versus BM EPCs under normoxia.

Relative enrichment of specific metabolites was analyzed by Liquid Chromatography-Mass Spectrometry (LC–MS) to define metabolic profiles and a heatmap for intermediates of glucose metabolism is presented in Fig. 2A. Hypoxic conditions resulted in reduced D-glucose and increased glucose-6-P (all p < 0.05), indicating a shift to greater glucose consumption and enhanced glycolysis (Fig. 2B). Levels of D-ribulose 5-P were also higher under hypoxia than under normoxia (p < 0.05; Fig. 2C), indicating enhanced pentose phosphate pathway (PPP) activity. By contrast, levels of citrate, isocitrate, succinate, fumarate, malate and oxaloacetate (OAA) were all lower under hypoxia than under normoxia (all p < 0.05, Fig. 2D), suggesting reduced TCA cycle activity under conditions of 1% O_2_.

### ^13^C glucose tracing

To ensure that the metabolites listed above originated from free glucose (rather than from gluconeogenesis, for example), stable isotope labeling experiments using ^13^C-labeled glucose were performed and isotopic enrichment quantified by High Pressure/Performance Liquid Chromatography-Q-Exactive-Mass Spectrometer (HPLC-QE-MS). No differences were found in ^13^C-labeled glycolytic intermediates, such as m + 3 or m + 6 iso-topologues of glucose 6-P, phosphoenolpyruvate or pyruvate, between the two conditions (Fig. 2E). However, there was a significant increase in ^13^C-labeled intermediates of the PPP, such as m + 6, m + 5 or m + 7 iso-topologues of 6-phosphogluconate, ribose 5-P and sedoheptulose 7-P (all p < 0.05), in hypoxic cells (Fig. 2F). Moreover, decreased ^13^C-labeling of TCA cycle intermediates, such as m + 2 iso-topologues of citrate, succinate and malate (all p < 0.05), was seen under hypoxia, suggesting a reduced contribution of glucose carbon to TCA cycle activity (Fig. 2G). Non-glucose-derived metabolites (m + 0 iso-topologues) of glycolysis, PPP and TCA cycle showed no significant differences between the two conditions. The above findings indicate greater flow of glucose carbon to the PPP and reduced flow to the TCA cycle under hypoxic conditions.

### Effects of glycolytic inhibition on BM EPCs proliferation

Key enzymes of pathways of glucose metabolism were inhibited and effects on cell stemness assessed.

**Figure 3:**
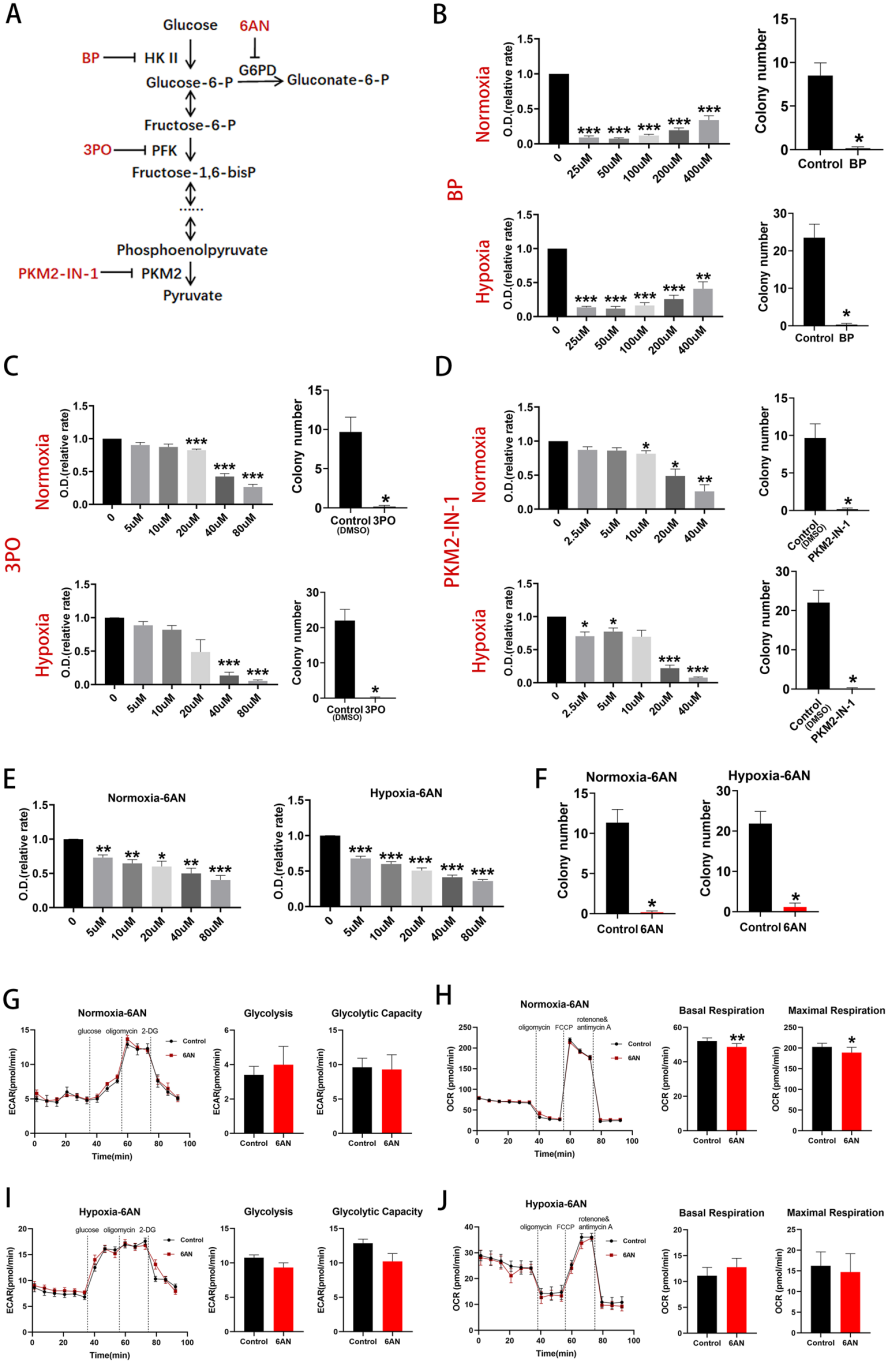
Inhibition of glycolysis reduced BM EPCs proliferation and inhibition of key enzymes of the PPP inhibited BM EPCs proliferation. (**A**) Schema for three key glycolytic enzymes, HK II, PFK and PKM, and their respective inhibitors, BP, 3PO and PKM2-IN-1. Schema for PPP enzyme, G6PD, and its respective inhibitor, 6AN. (**B**) Proliferation assay of BM EPCs treated with vehicle or various concentrations of BP under normoxia or hypoxia (n = 6). The number of EPC colonies in the presence of 100uM BP was significantly lower than that of the vehicle controls both under normoxia and under hypoxia (n = 6). (**C**) Proliferation assay of BM EPCs treated with vehicle or various concentration of 3PO under normoxia or hypoxia (n = 6). The number of BM EPCs colonies in the presence of 20uM 3PO was significantly lower than that of the vehicle controls both under normoxia and under hypoxia (n = 6). (**D**) Proliferation assay of BM EPCs treated with vehicle or various concentration of PKM2-IN-1 under normoxia or hypoxia (n = 6). The number of BM EPCs colonies in the presence of 10uM PKM2-IN-1 was significantly lower than that of the vehicle controls both under normoxia and hypoxia (n = 6). (**E**) Proliferation assay of BM EPCs treated with vehicle or various concentrations of 6AN under normoxia or hypoxia (n = 6). (**F**) The number BM EPCs colonies in the presence of 20uM 6AN was significantly lower than that of the vehicle controls both under normoxia and under hypoxia (n = 6). (**G**) Representative experiment showing ECAR of BM EPCs and comparison of glycolytic rate and capacity in BM EPCs treated with or without of 6AN under normoxia (n = 6). (**H**) Representative experiment showing OCR of BM EPCs and comparison of the OCR of basal and maximal respiration in the presence or absence of 6AN under normoxia. (**I**) Representative experiment showing ECAR of BM EPCs and comparison of glycolytic rate and capacity in the presence or absence of 6AN under hypoxia. (**J**) Representative experiment showing OCR of BM EPCs and comparison of basal and maximal respiration in the presence or absence of 6AN under hypoxia. Data are presented as Mean ± SEM. *p < 0.05; **p < 0.01; ***p < 0.001 versus vehicle control (with or without DMSO).

Bromopyruvate acid (BP) was used to inhibit hexokinase II (HK II), the 6-phosphofructo-2-kinase/fructose-2,6-biphosphatase (PFKFB3) inhibitor, compound 3-(3-pyridinyl)-1-(4-pyridinyl)-2-propen-1-one (3PO), to reduce fructose-2,6-bisphosphate (Fru-2,6-BP) levels and inhibit phosphofructokinase (PFK) and PKM2-IN-1 to inhibit pyruvate kinase (PKM2) (Fig. 3A)^13–15^. BP reduced EPC proliferation under both hypoxic and normoxic conditions and colony forming capacity was almost totally abolished by 100uM BP (Fig. 3B). 3PO reduced EPC proliferation under both hypoxic and normoxic conditions and colony forming capacity was almost totally abolished by 20uM 3PO (Fig. 3C). PKM2-IN-1 reduced EPC proliferation under both hypoxic and normoxic conditions and colony forming capacity was almost totally abolished by 10uM PKM2-IN-1 (Fig. 3D). These data demonstrate that glycolysis is essential for BM EPC proliferation.

### Effects of PPP inhibition on BM EPCs proliferation

PPP activity is vital for anabolic biosynthesis and anti-oxidant defense^16^ and depends on the diversion of glucose carbon away from the glycolytic pathway. Glucose-6-phosphate dehydrogenase (G6PD) is considered to be a key rate-controlling enzyme of the PPP and can be competitively inhibited by 6-AN (Fig. 3A)^17^. 6-AN reduced BM EPCs proliferation in a dose-dependent manner both under normoxia and under hypoxia (Fig. 3E) and colony formation in the presence of 20uM 6-AN was significantly lower than that of the vehicle controls (both p < 0.05; Fig. 3F).

6-AN did not affect glycolytic rate and capacity under normoxia (both p > 0.05; Fig. 3G) but reduced basal (p < 0.01) and maximal respiration (p < 0.05; Fig. 3H). No significant differences in glycolytic rate or capacity (both p > 0.05; Fig. 3I) or in basal or maximal respiration (p > 0.05, Fig. 3J) were seen with 6-AN under hypoxic conditions.

### Promotion of pyruvate oxidation reversed the effect of hypoxia in maintaining cell stemness

**Figure 4:**
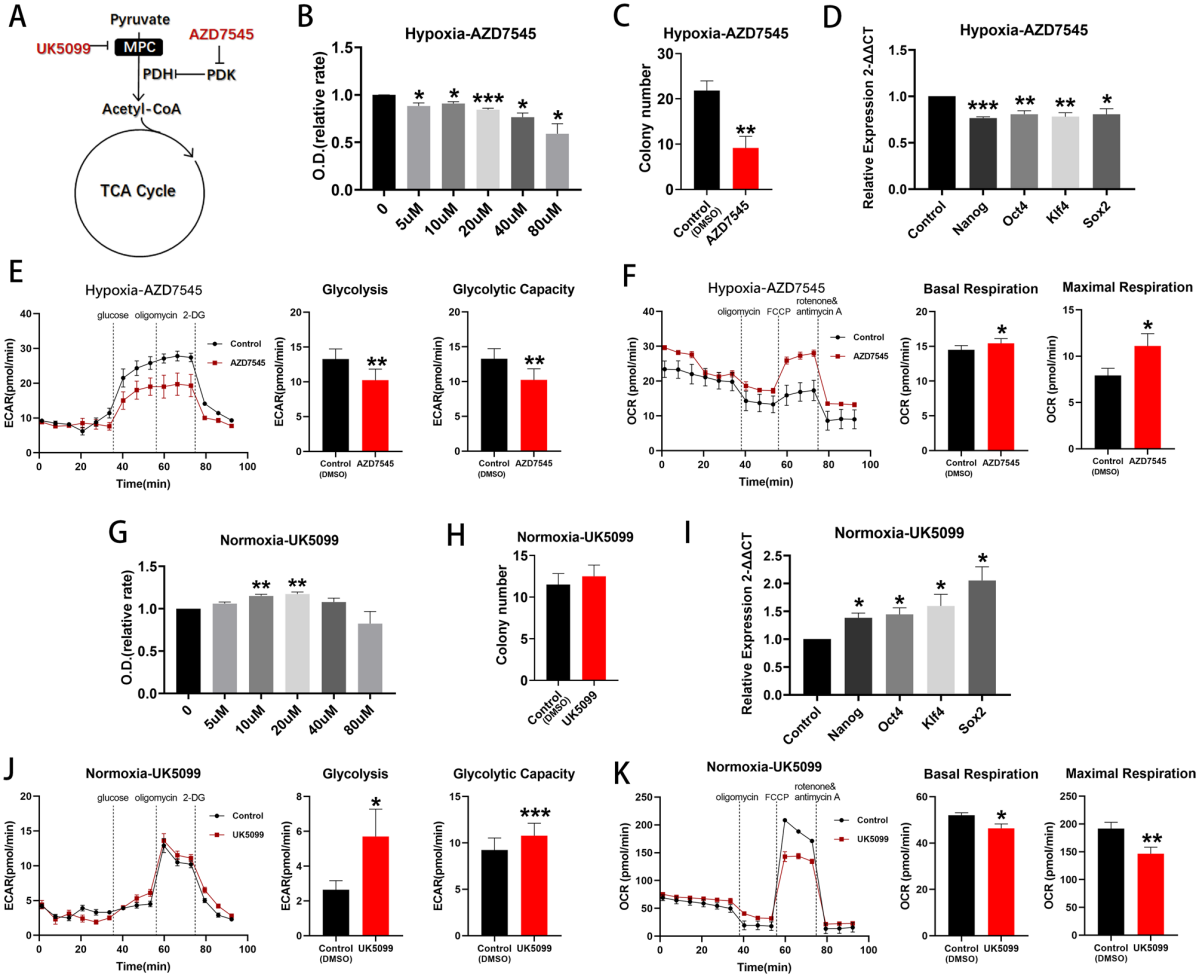
Effects of UK5099 and AZD7545 on BM EPCs stemness under normoxia and hypoxia, respectively. (**A**) Schema showing that the MPC inhibitor, UK5099, decreases pyruvate entry into the TCA cycle and that PDK inhibition by AZD7545 increases pyruvate oxidation. (**B**) Proliferation assay of BM EPCs treated with vehicle or various concentration of AZD7545 under hypoxia (n = 6). (C) Colony formation assay of BM EPCs treated with vehicle or 20uM AZD7545 under hypoxia (n = 6). (**D**) qRT-PCR showed lower expression of BM EPCs stemness markers in the presence of 20uM AZD7545 than with vehicle control under hypoxia (n = 5). (**E**) Representative experiment showing ECAR of BM EPCs and comparison of glycolytic rate and capacity in the presence or absence of AZD7545 under hypoxia (n = 6). **(F)** Representative experiment showing OCR of BM EPCs and comparison of basal and maximal respiration in the presence or absence of AZD7545 under hypoxia (n = 6). (**G**) Proliferation assay of BM EPCs treated with vehicle or various concentrations of UK5099 under normoxia (n = 6). (**H**) Colony formation assay of BM EPCs treated with 20uM UK5099 or vehicle under normoxia (n = 6). (**I**) qRT-PCR showed higher expression of BM EPCs stemness markers in the presence of 20uM UK5099 than with vehicle control under normoxia (n = 5). (**J**) Representative experiment showing ECAR of BM EPCs and comparison of the glycolytic rate and capacity in the presence or absence of UK5099 under normoxia (n = 6). (**K**) Representative experiment showing OCR of BM EPCs and comparison of basal and maximal respiration in the presence or absence of UK5099 under normoxia (n = 6). Data are presented as Mean ± SEM. *p < 0.05; **p < 0.01; ***p < 0.001 versus DMSO vehicle control.

Pyruvate dehydrogenase (PDH) catalyzes the irreversible oxidative decarboxylation of the glycolytic product, pyruvate, to the TCA cycle substrate, acetyl coenzyme A and flux through PDH, is negatively regulated by pyruvate dehydrogenase kinase (PDK) enzymes which may be inhibited by the compound, AZD7545 (Fig. 4A)^18,19^. Inhibition of PDK1, PDK2 and PDK3 by AZD7545, with the resulting stimulation of pyruvate oxidation^20,21^, suppressed cell proliferation (Fig. 4B), reduced colony formation (p < 0.01; Fig. 4C) and reduced expression of Nanog, Oct4, Klf4 and Sox2 (Fig. 4D) under hypoxic conditions. Glycolytic rate and capacity decreased (both p < 0.01, Fig. 4E) and basal and maximal respiration increased (both p < 0.05; Fig. 4F) under the same conditions. Use of AZD7545 under normoxic conditions produced the opposite results (Supplemental Fig. 1F–J). Overall, it appears that the promotion of pyruvate oxidation resulting from use of AZD7545 reversed the effects of hypoxia in maintaining stemness.

### Inhibition of pyruvate oxidation promoted cell proliferation under normoxia

The glycolytic product, pyruvate, is transported into the mitochondrion by the mitochondrial pyruvate carrier (MPC) for further oxidation. UK5099 is a potent inhibitor of MPC (Fig. 4A)^22^. Under normoxic conditions, 20 uM UK5099 promoted cell proliferation (p < 0.01; Fig. 4G) and, although colony formation was unaffected (p > 0.05; Fig. 4H), Nanog, Oct4, Klf4 and Sox2, were all upregulated (all p < 0.05; Fig. 4I). Moreover, glycolytic rate (p < 0.05) and capacity (p < 0.001) were both higher in the presence of UK5099 (Fig. 4J) and basal (p < 0.05) and maximal respiration rates (p < 0.01) were both lower (Fig. 4K). By contrast, UK5099 had little effect on cell proliferation, glycolytic rate and capacity, basal or maximal respiration under hypoxic conditions (Supplemental Fig. 1A–E). In combination, the above data suggest that inhibition of pyruvate oxidation promotes glycolysis, reduces mitochondrial respiration and increases BM EPCs proliferation under normoxia.

### Inhibition of the TCA cycle promoted cell proliferation under normoxia

**Figure 5:**
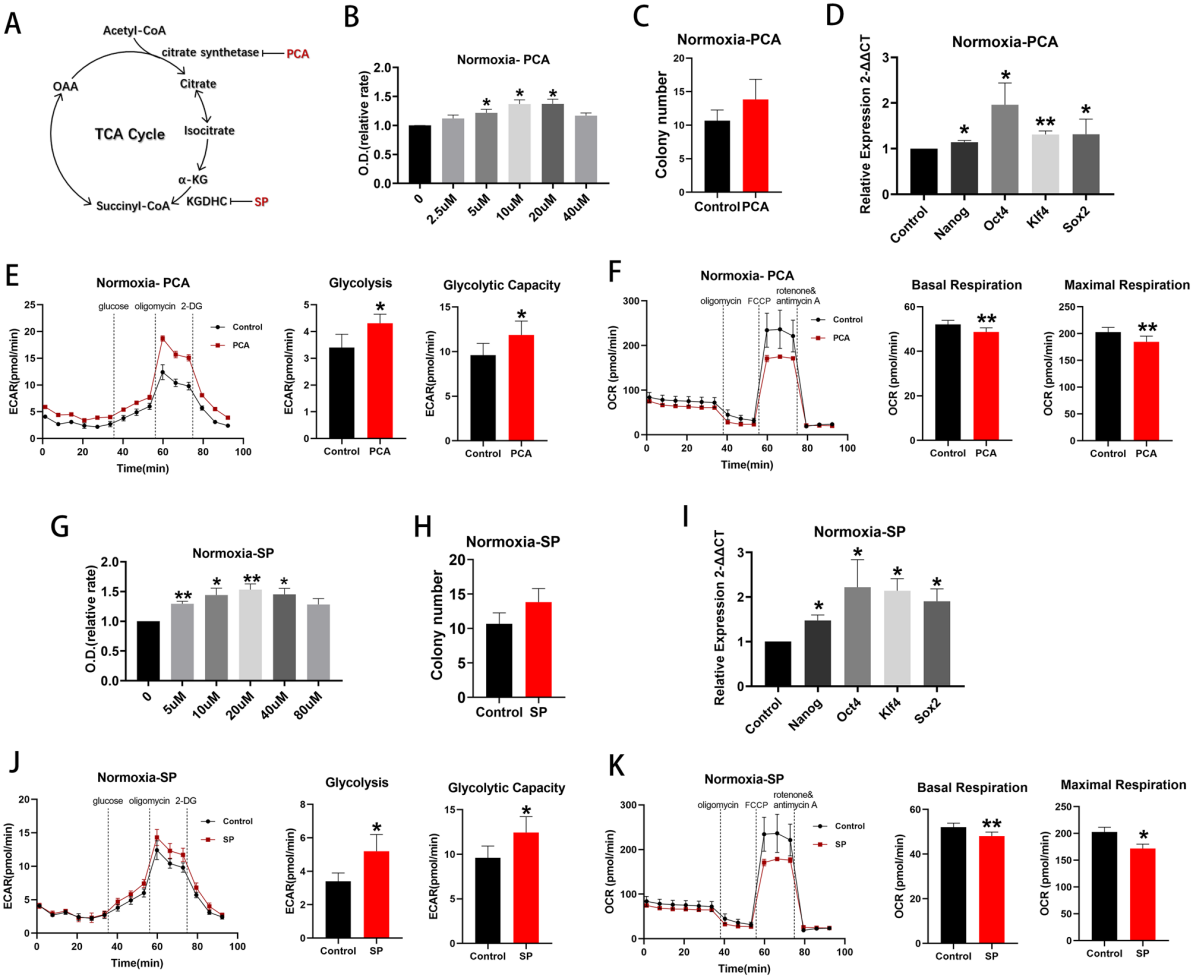
Inhibition of key TCA cycle enzymes increased cell proliferation under normoxia. (**A**) Schema for two enzymes, citrate synthase and KGDHC, in the TCA cycle and their respective inhibitors, PCA and SP. (**B**) Proliferation assay of BM EPCs treated with vehicle or various concentrations of PCA under normoxia (n = 6). (**C**) Colony formation assay of EPCs treated with vehicle or 10uM PCA under normoxia (n = 6). (**D**) qRT-PCR showed higher expression of BM EPCs stemness markers in the presence of 10uM PCA than with vehicle control under normoxia (n = 5). **(E**) Representative experiment showing ECAR of BM EPCs and comparison of glycolytic rate and capacity in the presence or absence of PCA under normoxia (n = 6). (**F**) Representative experiment showing OCR of BM EPCs and comparison of basal and maximal respiration in BM EPCs in the presence or absence of PCA under normoxia (n = 6). (**G**) Proliferation assay of BM EPCs treated with vehicle or various concentration of SP under normoxia (n = 6). (**H**) Colony formation assay of BM EPCs treated with vehicle or 20uM SP under normoxia (n = 6). (**I**) qRT-PCR showed higher expression of BM EPCs stemness markers in the presence of 20uM SP than with vehicle control under normoxia (n = 5). (**J**) Representative experiment showing ECAR of BM EPCs and comparison of glycolytic rate and capacity in the presence or absence of SP under normoxia (n = 6). (**K**) Representative experiment showing OCR of BM EPCs and comparison of basal and maximal respiration in the presence or absence of SP under normoxia (n = 6). Data are presented as Mean ± SEM. *p < 0.05; **p < 0.01 versus vehicle control (with or without DMSO).

Citrate synthase (CS), isocitrate dehydrogenase 3 (IDH3), and α-ketoglutarate dehydrogenase (KGDHC) all catalyze irreversible steps in the TCA cycle. Palmitoyl-coenzyme A (PCA) inhibits CS by interacting with the enzyme’s small domain^23,24^. Succinyl phosphonate trisodium salt (SP) inhibit KGDHC isoforms from muscle, bacteria, brain and cultured human fibroblasts (Fig. 5A)^25,26^. No chemical inhibitor of IDH3 was found.

TCA cycle inhibition by PCA promoted cell proliferation under conditions of normoxia (Fig. 5B), although colony formation was unaffected (p > 0.05, Fig. 5C). Expression of Nanog, Oct4, Klf4 and Sox2 was upregulated when PCA was present (all p < 0.05; Fig. 5D). Moreover, use of PCA increased glycolytic rate and capacity (both p < 0.05; Fig. 5E) and decreased basal (p < 0.05) and maximal respiration (p < 0.01; Fig. 5F) compared with controls. Very similar results were obtained with SP (Fig. 5G–K). Thus, inhibition of the TCA cycle increases flux through glycolysis and proliferation under normoxic conditions. By contrast, use of PCA or SP had little effect on cell proliferation, glycolytic rate and capacity, basal or maximal respiration under hypoxic conditions (Supplemental Fig. 2). The implication from these data is that the TCA cycle may already be maximally suppressed under hypoxic conditions and further inhibition has little detectable effect.

### Inhibition of the ETC

**Figure 6:**
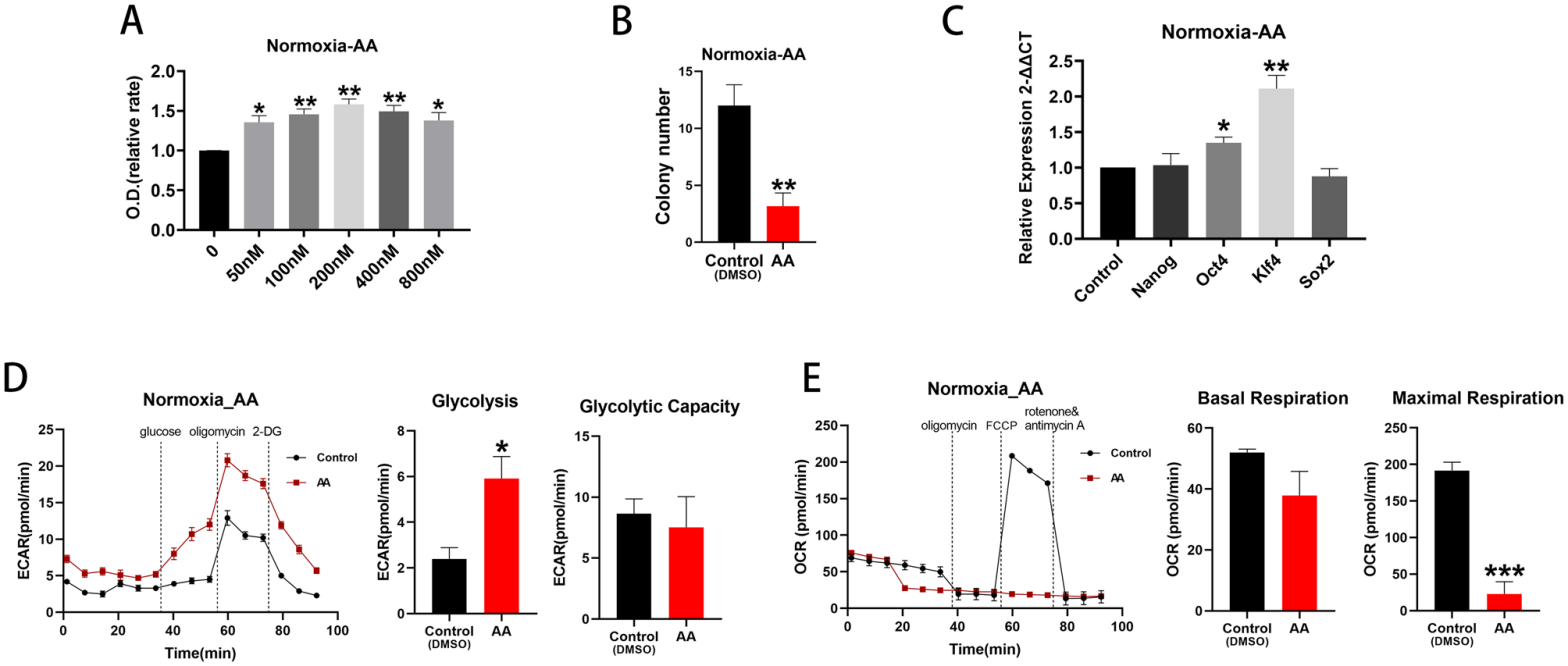
ETC inhibition promoted cell proliferation under normoxia. (**A**) Proliferation assay of BM EPCs treated with vehicle or various concentration of AA under normoxia (n = 6). (**B**) Colony formation assay of BM EPCs treated with vehicle or 200 nM AA under normoxia (n = 6). (**C**) qRT-PCR showed higher expression of BM EPCs stemness markers in the presence of 200 nM AA than with vehicle control under normoxia (n = 5). (**D**) Representative experiment showing ECAR of BM EPCs and comparison of glycolytic rate and capacity in the presence or absence of AA under normoxia (n = 6). (**E**) Representative experiment showing OCR of BM EPCs and comparison of basal and maximal respiration in the presence or absence of AA under normoxia (n = 6). Data are presented as Mean ± SEM. *p < 0.05; **p < 0.01; *** p < 0.001 versus DMSO vehicle control.

The complex III inhibitor, antimycin A (AA) was used to block the ETC^27^. Use of AA promoted BM EPCs proliferation (Fig. 6A) under normoxic conditions, although there was a significant reduction in colony formation (p < 0.05; Fig. 6B). Production of Oct4 and Klf4 mRNA (both p < 0.05; Fig. 6C) and glycolytic rate (p < 0.05; Fig. 6D) were all stimulated and maximal respiration (p < 0.001; Fig. 6E) reduced compared with controls. By contrast, use of AA had little effect on cell proliferation, glycolytic rate and capacity, basal or maximal respiration under hypoxic conditions (Supplemental Fig. 3).

### Adenosine triphosphate (ATP) production

Although a significant suppression of mitochondrial ATP production under-hypoxia (p < 0.001) was found, total cellular ATP production (p > 0.05) was unaffected, compared with normoxia (Supplemental Fig. 4A). Pharmacological manipulation of ATP level was then investigated, although inhibitors of glycolysis or the PPP could not be tested, since they had been shown to suppress BM EPCs growth. UK5099 reduced mitochondrial ATP production (p < 0.05) but did not affect total cellular ATP either under normoxia or hypoxia (p > 0.05, Supplemental Fig. 4B). AZD7545 reduced mitochondrial ATP under noxmoxia (p < 0.05) but did not affect cellular ATP either under normoxia or hypoxia (both p > 0.05; Supplemental Fig. 4C). Similarly, the two TCA cycle inhibitors, PCA and SP, reduced mitochondrial ATP under normoxia (all p < 0.05) but did not affect cellular ATP level either under normoxia or hypoxia (all p > 0.05; Supplemental Fig. 4D,E). Use of AA also reduced mitochondrial ATP level under both normoxic (p < 0.001) and hypoxic (p < 0.05) conditions but had no effect on total cellular ATP level under either condition (p > 0.05; Supplemental Fig. 4F).

## Discussion

The current study describes reprogramming of pathways related to glucose metabolism in human BM-derived EPCs under hypoxic conditions of 1% O_2_ and demonstrates the relationship between metabolic reprogramming and stemness. Hypoxia was found to increase colony formation and expression of stemness markers compared with normoxia, findings consistent with previous studies^4^. Thus, hypoxic conditions are of benefit for maintenance of stemness in cultured BM EPCs. Experiments using the Seahorse XFe96 Flux Analyzer revealed enhanced glycolytic rate and capacity but reduced basal and maximal respiration under hypoxic conditions. Hypoxia may induce a metabolic switch away from mitochondrial respiration to glycolysis.

Metabolic profiles established by LC–MS were characterized by increased glycolytic and PPP intermediates (glucose-6-P and D-ribulose 5-P) and reduced TCA cycle intermediates (citrate, isocitrate, succinate, fumarate, malate and oxaloacetate) under hypoxic conditions. Increased flow of label from ^13^C- glucose was found in PPP intermediates and decreased flow to TCA cycle intermediates during hypoxia. Thus, hypoxia caused greater flow of carbon from glucose to the PPP at the expense of that to the TCA cycle.

Many rapidly-proliferating cell-types have been shown to undergo similar metabolic shifts. For example, stimulation of glycolysis via hypoxia or inhibition of mitochondrial respiration in pluripotent stem cells promotes stemness^28,29^ while inhibition of glycolysis halts proliferation and precipitates cell death^30^. Hypoxia has also been shown to induce a metabolic switch between the PPP and glycolysis in glioma stem-like cells^31^. Glycolysis is far less efficient than mitochondrial respiration in terms of ATP-production, producing only 2ATP/glucose compared with 30-32ATP/glucose from full mitochondrial oxidation. Rapidly-proliferating cells are likely to have high requirements for ATP, as well as for carbon, nitrogen and hydrogen to support biosynthetic activity, which makes a shift towards the less efficient glycolysis pathway difficult to interpret^32,33^. However, an exploration of the metabolic cost of synthesizing enzymes for fermentation versus aerobic respiration in E. coli found that energy expenditure in synthesizing proteins for mitochondrial respiration outweighed the energy gain from more efficient ATP-production. Thus, it may make more sense for fast-growing cells to utilize glycolysis for efficient cell growth and proliferation^34^.

Inhibitors of glucose metabolism were used to assess the impact of the dependent pathways on cell stemness. Inhibition of the glycolytic enzymes, HK II, PFK and PKM, by BP, 3PO and PKM2-IN-1, respectively, reduced growth rates of BM EPCs under both hypoxia and normoxia. BP has previously been shown to inhibit ovarian cancer growth and to induce apoptosis of hepatocellular carcinoma cells and human breast cancer cells^35–37^. PKM2-IN-1 reduced glucose uptake and lactate production in esophageal and hepatocellular carcinoma cells^38,39^ and 3PO inhibited growth of lung cancer, breast cancer and promyelocytic leukemia cells^14^. The above findings demonstrate the importance of glycolysis for proliferation of tumor cells.

The PPP produces ribose-5-phosphate for DNA/RNA synthesis and NADPH for fatty acid synthesis and branches off from glycolysis during the preparatory phase. Shunting of glucose carbon through the PPP balances requirements for catabolic generation of ATP and reducing cofactors with production of biosynthetic substrates to meet anabolic needs^40,41^. Inhibition of the PPP enzyme, G6PD, by 6-AN, inhibited proliferation of glioblastoma cells and colony formation in embryonic stem cells^17,42^. The current study also found that 6-AN reduced EPC proliferation, demonstrating a critical role of the PPP in EPC growth.

Pyruvate is central to glucose metabolism, whether through lactate fermentation or the TCA cycle^43^, and also to synthesize lipids and amino acids. Use of the PDK inhibitor, AZD7545, to promote pyruvate oxidation has been shown to inhibit the growth of melanoma^44^ and suppress proliferation of activated primary human CD4 + T cells and primary mouse T cells^45^. The current study found that AZD7545 decreased glycolytic rate and capacity, increased basal and maximal respiration and reduced EPC proliferation under hypoxic conditions. Thus, promotion of pyruvate oxidation appears to reverse the effect of hypoxia in promoting cell proliferation.

By contrast, use of the MPC blocker, UK5099, has previously been shown to induce metabolic reprogramming and enhance stem-like properties in human prostate cancer cells^46^. Similarly, ovarian cancer cells switched to aerobic glycolysis with reduced ATP production and became more migratory and resistant to chemotherapy and radiotherapy in the presence of UK5099^47^. The current study found increased glycolytic rate and capacity, decreased basal and maximal respiration and enhanced BM EPCs proliferation under normoxic but not under hypoxic conditions when UK5099 was present.

The TCA cycle inhibitors, PCA and SP, increased glycolytic rate and capacity, decreased basal and maximal respiration and enhanced BM EPCs proliferation under normoxia. However, these inhibitors had little effect under hypoxia, a state in which the TCA cycle is suppressed. Similarly, the complex III inhibitor, AA, increased glycolysis, decreased maximal respiration, increased cell proliferation and stem cell marker mRNA levels under normoxia but not under hypoxia.

Metabolism has traditionally be regarded as fulfilling three roles: ATP-production for energy-requiring processes; production of precursors for anabolism and production of intermediates for enzyme-catalyzed reactions^48^. The first of these roles, ATP production, was found to be suppressed on a mitochondrial level but unaffected on a cellular level by hypoxic conditions. Similar results were found by use of inhibitors. Inhibition of pyruvate oxidation by UK5099 reduced mitochondrial, but not cellular, ATP under both normoxia and hypoxia. Inhibition of the TCA cycle by PCA or SP also reduced mitochondrial ATP under noxmoxic conditions but did not affect cellular ATP either under normoxia or hypoxia. The ETC inhibitor, AA, reduced mitochondrial ATP under both normoxia and hypoxia but had no effect on total ATP. The import of these data is that energy production does not have much significance in cell proliferation and production of biosynthetic and reaction intermediates may have greater responsibility for metabolic reprogramming.

Taken together, we have shown that hypoxic conditions of 1% O_2_ maintain BM EPCs stemness and induce metabolic reprogramming, manifested as increased glycolysis and PPP and decreased TCA cycle and mitochondrial respiration. The current study has demonstrated that inhibition of glycolysis or the PPP or promotion of mitochondrial respiration resulted in a reversal of the effect of hypoxia in maintaining cell stemness. By contrast, inhibition of pyruvate oxidation, the TCA cycle or the ETC promoted BM EPCs proliferation under normoxic conditions mimicking hypoxic conditions. In conclusion, hypoxia induced glucose metabolic reprogramming, and such pattern of metabolic reprogramming maintains the stemness of BM EPCs, and artificial manipulation of cell metabolism can be an effective way for regulating the stemness of BM EPCs.

We acknowledge some limitations to the current study. Firstly, we tested the effects of glucose metabolism on EPCs stemness by intervention at different stages of glucose metabolic pathways. However, this is just a preliminary blueprint study, the underlying molecular mechanisms of each specific enzyme involved in hypoxia-induced metabolic reprogramming and maintenance of BM EPCs stemness remain to be clarified. Secondly, we only detected glucose metabolism, and the other metabolism, such as amino acid metabolism and lipid metabolism, are worthy of future study on their relationship with EPCs stemness. Thirdly, in vivo experiments are required to clarify whether hypoxia or drug-induced enhancement of BM EPCs stemness has application to clinical transplantation therapy.

## Methods

### Cell culture

This study was approved by the Ethical Committee of Zhujiang Hospital, Southern Medical University (No. 2019-SJWK-007, 2019-3-21, Guangdong, China) and all methods were performed in accordance with the relevant guidelines and regulations along with the Declaration of Helsinki. All cell donors gave written informed consent. BM EPCs were isolated as described previously by our and other labs^3,4,49–51^. Briefly, BM was extracted from eight healthy donors (4 males: 34, 45, 45 and 58 years old; 4 females: 34, 42, 45 and 66 years old) by puncture of the posterior superior iliac crest and BM mononuclear cells isolated by centrifugation through a density gradient (Ficoll-Paque Plus, Pharmacia). Mononuclear cells were seeded onto fibronectin-coated (354,008, BD Pharmingen) plastic dishes (1 × 10^5^ cells/cm^2^) and cultured in EC basal medium-2 (EBM-2, CC-3156, Lonza) with EGM-2 MV SingleQuots (CC-4176, Lonza) at 37 °C with either 1% O_2_/5% CO_2_/94% N_2_ (hypoxia, HERAcell VIOS 160i, Thermo, USA) or 5% CO_2_/95% ambient air (~ 20% O_2_, normoxia, Thermo 371), respectively. After 4 days, non-adherent cells were discarded by removing the medium. At 80% confluence, cells were detached with 0.05% trypsin/0.01% EDTA and sub-cultured. Cell morphology was observed under a Type CK2 phase-contrast microscope (Olympus). To identify EPCs, cell binding of UEA-1 lectin and endocytosis of acetylated low-density lipoprotein (Dil-Ac-LDL), two characteristic features of endothelial lineage cells, were detected as previously described^2,52^.

The following inhibitors were used: BP (Sigma, 1113-59-3), 3PO (MCE, HY-19824), PKM2-IN-1 (MCE, HY-103617), 6-AN (Sigma, 329-89-5), UK5099 (MCE, HY-15475), AZD7545 (Selleck Chemicals, s7517), PCA (Sigma, 188174-64-3), SP (MCE, HY-12688A) and AA (Abcam, Ab141904). 3PO, PKM2-IN-1, UK5099, AZD7545 and AA were dissolved in EBM-2 with DMSO. BP, 6AN, PCA and SP were dissolved in EBM-2 without DMSO.

### Colony formation assay

BM EPCs were seeded onto 6-well plates (100 cells per well) and incubated under normoxia/ hypoxia for 24 h. 30 μL of different concentrations of inhibitors or vehicles (with or without DMSO) were added and incubated under normoxia / hypoxia conditions for 8 days. Cells were washed twice with phosphate buffered saline (PBS) and fixed with 1 mL 4% paraformaldehyde for 15 min and incubated with 1 mL 0.1% crystal violet for 20 min. Excess crystal violet was washed off with dH_2_O and dishes allowed to dry. Photographs were taken and the colonies containing > 50 individual cells counted.

### Quantitative real-time PCR (qRT-PCR)

Total RNA was isolated using Trizol reagent (Invitrogen, USA) and cDNA synthesized using Evo M-MLV RT Kit with gDNA Clean for qPCR (Accurate Biology AG, hunan, China). qRT-PCR was performed with SYBR Green Premix Pro Taq HS qPCR Kit (Accurate Biology AG, hunan, China) on an ABI QuantStudio™ 3 System Real-Time PCR System (Applied Biosystems, USA). Primers for genes encoding β-actin, Nanog, OCT4, Sox2 and KLF4^53,54^ were purchased from Ige biotechnology ltd. Primer sequences were as follows: Human β-actin: forward 5′-AGCGAGCATCCCCCAAAGTT-3′; reverse 5′-GGGCACGAAGGCTCATCATT-3′; Human Nanog: forward 5′-AATGTCTTCTGCTGAGATGCCT-3′; reverse 5′-GAAGTGGGTTGTTTGCCTTTGG -3′; Human OCT4: forward 5′-TGGGAAGGTATTCAGCCAAACG -3′; reverse 5′- CACGAGGGTTTCTGCTTTGCAT -3′; Human Sox2: forward 5′- CATCACCCACAGCAAATGAC -3′; reverse 5′- CAAAGCTCCTACCGTACCACT -3′; Human Klf4: forward 5′- CATCTCAAGGCACACCTGCGAA -3′; reverse 5′-TCGGTCGCATTTTTGGCACTGG -3′. qRT-PCR was performed in duplicate in a 20-μl reaction volume. Fold differences were determined using β-actin as an internal reference by the 2^−∆∆Ct^ method.

### Tube formation assay

The tube formation assay was carried out to estimate the angiogenic capacity of BM EPCs. Briefly, EPCs (3 × 10^4^cells) were seeded onto 96-well plates coated with growth factor-reduced matrigel (70 μl/well; BD Biocoat) and cultured in EBM-2 at 37 °C under hypoxia or normoxia. After incubation for 8 h, tubes in each well were visualized under an inverted microscope, photographed at 10 × magnification, and analyzed using the Angiogenesis Analyzer for ImageJ software.

### Extracellular flux analysis

Kinetic metabolic profiling was performed in real time by the fully integrated 96-well Seahorse Bioscience Extracellular XFe96 Flux Analyzer (Seahorse Bioscience, North Billerica, MA). Extracellular acidification rate (ECAR) to measure glycolytic capacity was determined using the XF Glycolysis Stress Test Kit (Seahorse Bioscience, North Billerica, MA) and oxygen consumption rate (OCR) to reflect mitochondrial respiration using the XF Cell Mito Stress Test Kit (Seahorse Bioscience, North Billerica, MA).

BM EPCs were prepared by seeding 2 × 10^4^ cells per well into a XFe96 Microplate (Seahorse Bioscience, North Billerica, MA) and incubating under normoxia/ hypoxia for 24 h. Cells were washed twice with PBS and incubated in pH 7.4 Seahorse XF Assay Medium for 1 h at 37 °C without CO_2_. Inhibitors or vehicle were added to through hole A of the feeding hole. Metabolic profiles were determined by adding glucose (10 mM), oligomycin (2 μM) and 2-DG (50 mM) for ECAR; oligomycin (2 μM), carbonyl cyanide 4-(trifluoromethoxy) phenylhydrazone (FCCP, 2 μM), rotenone/antimycin A (1 μM) for OCR (all Seahorse Bioscience, North Billerica, MA).

### LC–MS conditions for metabolite detection

LC–MS/MS analyses were performed using an ExionLC™ AD system (SCIEX) coupled with a QTRAP^®^ 6500 + mass spectrometer (SCIEX). 1 × 10^7^ BM EPCs (50 μL). Pre-chilled 80% methanol (200 μL) was vortex mixed and sonicated for 6 min. Samples were incubated on ice for 5 min before centrifugation at 15,000 *rpm*, 4 °C for 10 min. Supernatant was diluted to the final concentration by addition of LC–MS grade water containing 53% methanol. Samples were transferred to a fresh Eppendorf tube and centrifuged at 15,000 *g* at 4 °C for 20 min. Filtrate was injected into the LC–MS/MS system via a Waters Atlantis premier BEH C18 Column (2.1 × 100 mm) using a 10-min linear gradient at a flow rate of 0.3 mL/min. Eluents were A: 0.5% formic acid/30 mM ammonium formate and B: 0.5% formic acid–methanol). The solvent gradient was set as follows: 0% B for 2 min; 0–100% B for 2.01 min; 100% B for 6 min; 100–0% B for 8.01 min; 0% B for 10 min. QTRAP^®^ 6500 + mass spectrometer was operated in positive polarity mode with Curtain Gas of 35 psi, Collision Gas of Medium, IonSpray Voltage of 4500 V, Temperature of 550 °C, Ion Source Gas 1:60, Ion Source Gas 2: 60 and in negative polarity mode with Curtain Gas of 35 psi, Collision Gas of Medium, IonSpray Voltage of − 4500 V, Temperature of 550 °C, Ion Source Gas 1:60, Ion Source Gas 2:60. Detection of experimental samples used MRM (Multiple Reaction Monitoring), based on novogene in-house database. Q3 was used for metabolite quantification. Q1, Q3, RT (retention time), DP (declustering potential) and CE (collision energy) were used for metabolite identification. Data files generated by HPLC–MS/MS were processed using SCIEX OS Version 1.4 to integrate and correct the peak.

### ^13^C tracing by HPLC-QE-MS

Cells were cultured under normoxia/ hypoxia and culture medium replaced with labeling medium containing 2 g/L [U6]-^13^C-glucose (Sigma) followed by incubation for 24 h. 250 μL of water was added to 1 × 10^5^ EPCs followed by vortex mixing for 30 s. Samples were pre-cooled in dry ice, freeze-thawed three times in liquid nitrogen, vortexed for 30 s and sonicated for 10 min in an ice-water bath. 750 μL of methanol was added to the sample solution with vortex mixing and vibration at 4 °C for 15 min, followed by incubation at − 40 °C for 1 h. Samples were centrifuged at 12,000 rpm (RCF = 13,800(× *g*), R = 8.6 cm) for 15 min at 4 °C. 800 μL of the clear supernatant was collected and dried in a vacuum concentrator. Residue was reconstituted with ultrapure water according to the cell count, vortexed and sonicated for 10 min in an ice-water bath before filtration through the centrifuge tube filter. Supernatant were transferred to inserts in injection vials for HPLC-QE-MS analysis.

HPLC separation was carried out using a Thermo Scientific Dionex ICS-6000 HPLC System (Thermo Scientific) equipped with Dionex IonPac AS11-HC (2 × 250 mm) and AG11-HC (2 mm × 50 mm) columns. Mobile phase A consisted of 100 mM NaOH in water and phase C of water. An additional pumping system was used to supply the solvent (2 mM acetic acid in methanol) and solvent mixed with eluent before loading of the ion source (flow rate of 0.15 mL/min). The column temperature was set at 30 °C, that of the auto-sampler at 4 °C and the injection volume was 5 μL. The QE mass spectrometer was used to acquire MS spectra in full MS mode with acquisition software (Xcalibur 4.0.27, Thermo) to continuously evaluate the full scan MS spectrum.

### Proliferation assay

BM EPCs were seeded onto 96-well plates (4000 cells per well) and incubated under normoxia or hypoxia for 1 day in a final volume of 90 μL/well. 10 μL of inhibitor or vehicle (with or without DMSO) were added to each well and incubated under conditions of normoxia/hypoxia for 3 days. 10 μL Cell Counting Kit-8 (Dojindo, Japan) mixture was added and the plate incubated for 2 h before measurement of absorbance at 450 nm by microplate reader (Thermo FC).

### ATP measurement

Total ATP was measured using ATP Assay Kit (Beyotime) according to the manufacturer’s instructions. Briefly, 5 × 10^5^ BM EPCs were lysed with 200 μL lysis buffer and ultrasonicated after washing twice with PBS. The lysate was centrifuged at 12 000 × *g* for 15 min at 4 °C. 20 uL per well supernatant was added to a microplate together with 100 uL ATP detection solution. Chemiluminescence was detected by a luminescence reader (Synergy H1). Mitochondrial ATP was measured by using the XF Cell Mito Stress Test Kit (Seahorse Bioscience, North Billerica, MA) by subtracting the rate measurement before oligomycin addition from that after oligomycin.

### Statistical analysis

Data are presented as mean ± SEM and paired Student’s t-test was used to compare parameter data, Wilcoxon test for nonparametric data and ANOVA for repeated measurement data. A value of p < 0.05 was considered significant.

### Ethics approval and consent to participate

This study was approved by the Ethical Committee of Zhujiang Hospital, Southern Medical University (No. 2019-SJWK-007, 2019-3-21, Guangdong, China) and performed in accordance with the Declaration of Helsinki.

## Supplementary Information

Supplementary Figures.

## Data Availability

All data and materials generated and analyzed during the present study are available from the corresponding author on reasonable request.

## Abbreviations

3PO: 3-(3-Pyridinyl)-1-(4-pyridinyl)-2-propen-1-one
6-AN: 6-Aminonicotinamide
α-KG: α-Ketoglutaric acid
AA: Antimycin A
ATP: Adenosine triphosphate
BM: Bone marrow
BP: Bromopyruvic acid
DMSO: Dimethyl sulfoxide
ECAR: Extracellular acidification rate
BM EPCs: Bone marrow-derived endothelial progenitor cells
ETC: Mitochondrial electron transport chain
FCCP: Carbonyl cyanide-4 (trifluoromethoxy) Phenylhydrazone
Fru-2,6-BP: Fructose-2,6-bisphosphate
G6PD: Glucose-6-phosphate dehydrogenase
HK II: Hexokinase II
HPLC-QE-MS: High pressure/performance liquid chromatography-Q-exactive-mass spectrometer
KGDHC: α-Ketoglutarate dehydrogenase
LC–MS: Liquid chromatography-mass spectrometry
MPC: Mitochondrial pyruvate carrier
NADH: Nicotinamide adenine dinucleotide
NADPH: Nicotinamide adenine dinucleotide phosphate
OAA: Oxaloacetate
OCR: Oxygen consumption rate
PBS: Phosphate buffered saline
PCA: Palmitoyl coenzyme A lithium salt
PDH: Pyruvate dehydrogenase
PDK: Pyruvate dehydrogenase kinase
PFK: Phosphofructokinase
PFKFB3: 6-Phosphofructo-2-kinase/fructose-2,6-biphosphatase
PKM: Pyruvate kinase
PPP: Pentose phosphate pathway
SP: Succinyl phosphonate trisodium salt
TCA: Tricarboxylic acid
