# Supplementary material for: Hypoxia-induced reprogramming of glucose-dependent metabolic pathways maintains the stemness of human bone marrow-derived endothelial progenitor cells
Source: Sci Rep. 2023 May 31;13:8776. doi: 10.1038/s41598-023-36007-5 (PMC10232473; doi:10.1038/s41598-023-36007-5)
Supplement: Supplementary file 1 — Supplementary Figures. [file 41598_2023_36007_MOESM1_ESM.docx]

**Supplemental Figures**


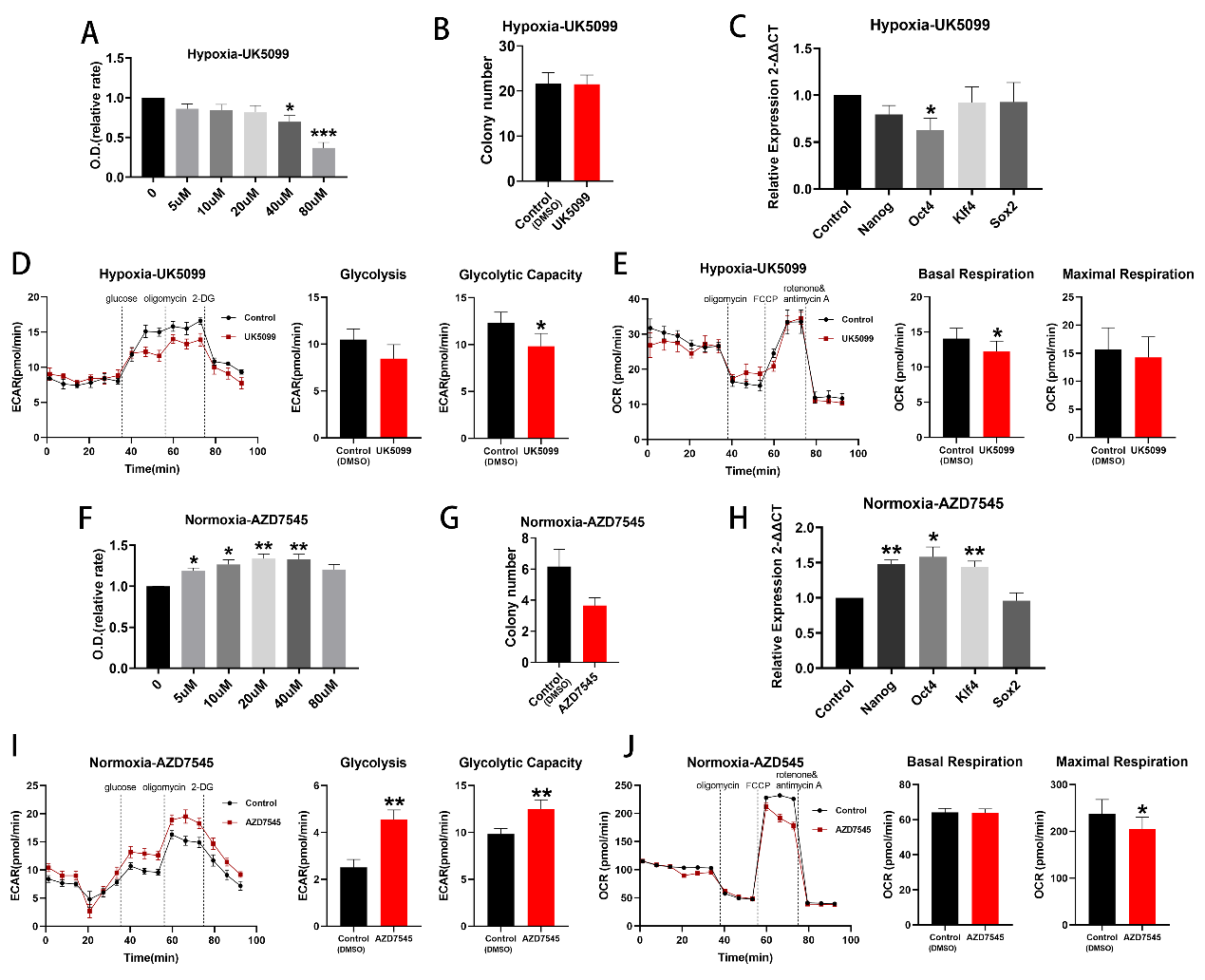


**Figure 1.** Effects of UK5099 and AZD7545 on BM EPCs stemness under hypoxia and normoxia, respectively. **(A)** Proliferation assay of EPCs treated with vehicle or various concentrations of UK5099 under hypoxia (n = 6). **(B)** Colony formation assay of EPCs treated with vehicle or 20uM UK5099 under hypoxia (n = 6). **(C)** qRT-PCR showing expression of EPC stemness markers after treatment with vehicle or 20uM UK5099 under hypoxia (n=5). **(D)** Representative experiment showing ECAR of EPCs and comparison of glycolytic rate and capacity in the presence and absence of UK5099 under hypoxia (n = 6). **(E)** Representative experiment showing OCR of EPCs and comparison of basal and maximal respiration in the presence and absence of UK5099 under hypoxia (n = 6). **(F)** Proliferation assay of EPCs treated with vehicle or various concentrations of AZD7545 under normoxia (n = 6). **(G)** Colony formation assay of EPCs treated with vehicle or 20uM AZD7545 under normoxia (n = 6). **(H)** qRT-PCR showing expression of EPC stemness markers after treatment with 20uM AZD7545 under normoxia. **(I)** Representative experiment showing ECAR of EPCs and comparison of glycolytic rate and capacity in the presence and absence of AZD7545 under normoxia (n = 6). **(J)** Representative experiment showing OCR of EPCs and comparison of basal and maximal respiration in the presence and absence of AZD7545 under normoxia (n = 6). Data are presented as Mean ± SEM. *p < 0.05; **p < 0.01 versus DMSO vehicle control.

**
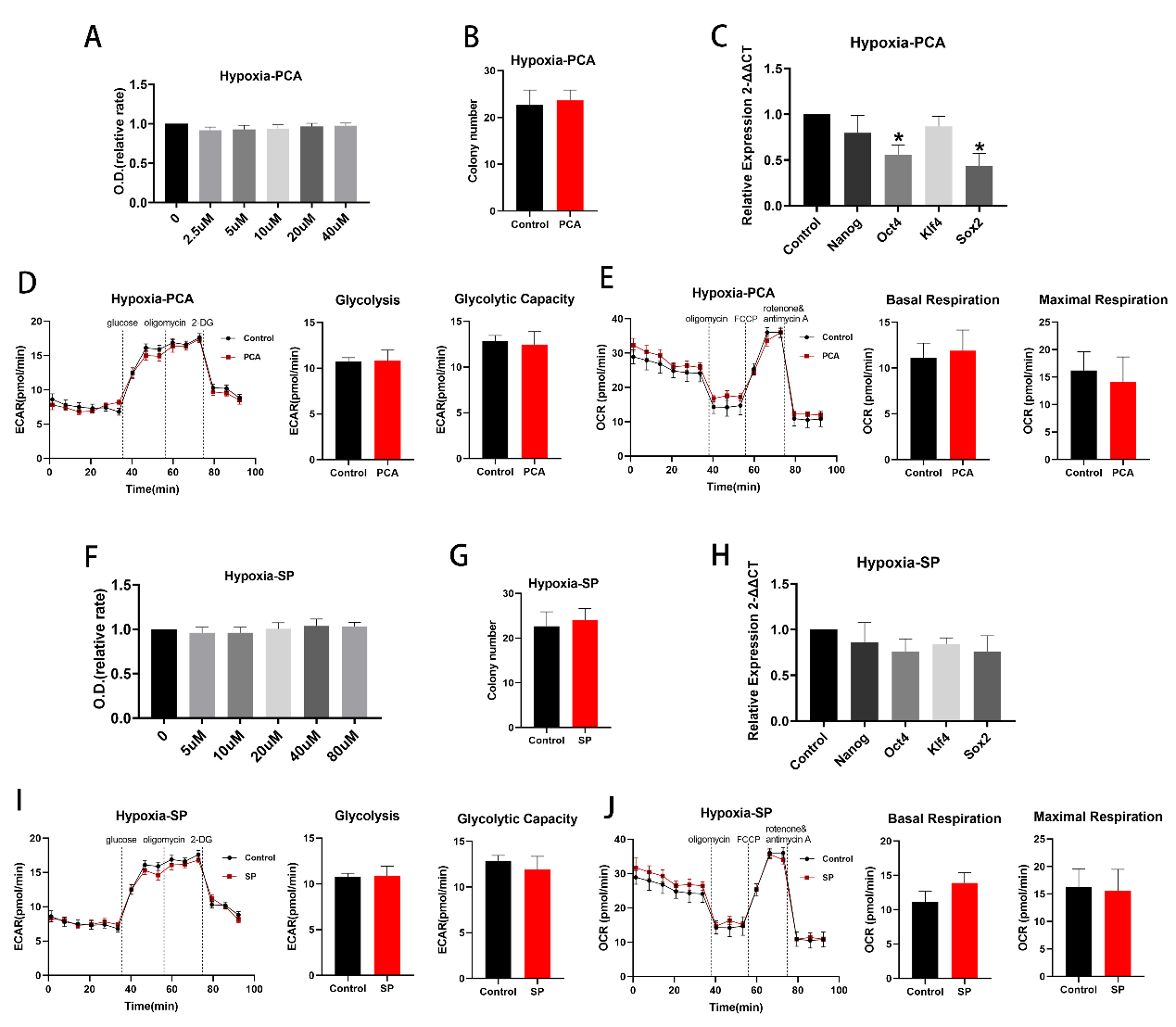
**

**Figure 2.** Effects of PCA and SP on BM EPCs stemness under hypoxia. **(A)** Proliferation assay of EPCs treated with vehicle or various concentrations of PCA under hypoxia (n = 6). **(B)** Colony formation assay of EPCs treated with vehicle or 10uM PCA under hypoxia (n = 6). **(C)** qRT-PCR showing expression of EPC stemness markers in the presence or absence of 10uM PCA under hypoxia (n=5). **(D)** Representative experiment showing ECAR of EPCs and comparison of glycolytic rate and capacity in the presence or absence of PCA under hypoxia (n = 6). **(E)** Representative experiment showing OCR of EPCs and comparison of basal and maximal respiration in the presence or absence of PCA under hypoxia (n = 6). **(F)** Proliferation assay of EPCs treated with vehicle or various concentration of SP under hypoxia (n = 6). **(G)** Colony formation assay of EPCs treated with vehicle or 20uM SP under hypoxia (n = 6). **(H)** qRT-PCR showing expression of EPC stemness markers after treatment with vehicle or 20uM SP under hypoxia (n=5). **(I)** Representative experiment showing ECAR of EPCs and comparison of glycolytic rate and capacity in the presence or absence of SP under hypoxia (n = 6). **(J)** Representative experiment showing OCR of EPCs and comparison of basal and maximal respiration in the presence or absence of SP under hypoxia (n = 6). Data are presented as Mean ± SEM. *p < 0.05 versus vehicle control (with or without DMSO).


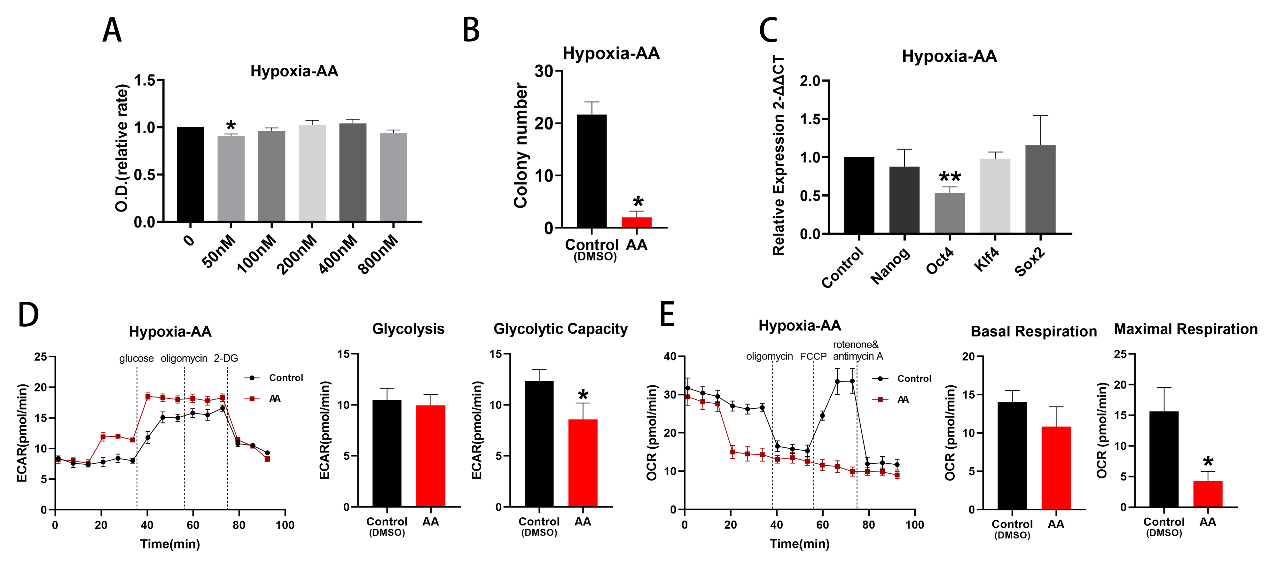


**Figure 3.** Effects of AA on BM EPCs stemness under hypoxia. **(A)** Proliferation assay of EPCs treated with vehicle or various concentrations of AA under hypoxia (n = 6). **(B)** Colony formation assay of EPCs treated with vehicle or 200nM AA under hypoxia (n = 6). **(C)** qRT-PCR showing expression of EPC stemness markers after treatment with vehicle or 200nM AA under hypoxia (n=5). **(D)** Representative experiment showing ECAR of EPCs and comparison of glycolytic rate and capacity in the presence or absence of AA under hypoxia (n = 6). **(E)** Representative experiment showing OCR of EPCs and comparison of basal and maximal respiration in the presence or absence of AA under hypoxia (n = 6). Data are presented as Mean ± SEM. *p < 0.05; **p < 0.01 versus DMSO vehicle control.


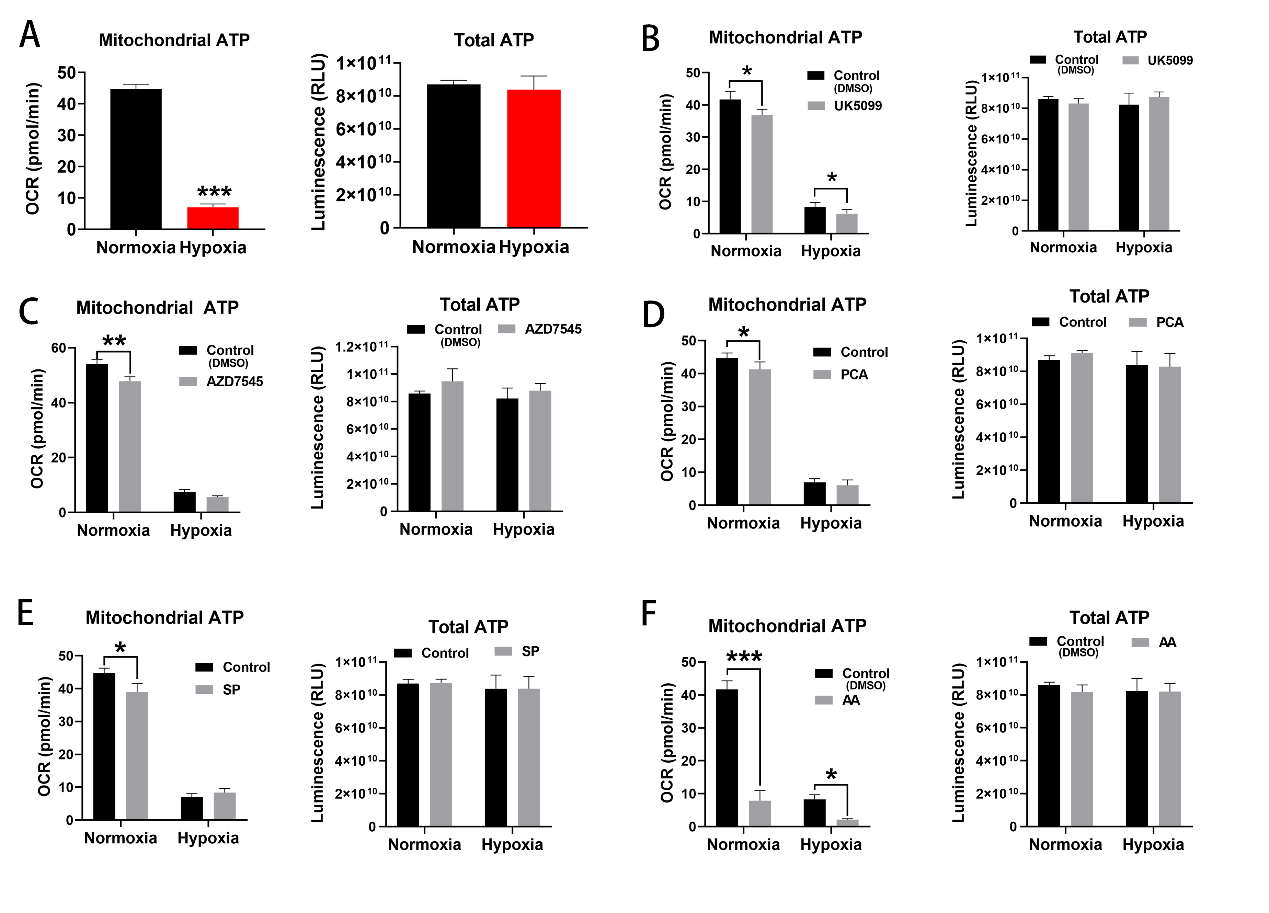


**Figure 4.** Hypoxia- or inhibitor-induced metabolic shift did not affect total ATP production. **(A)** Comparison of mitochondrial and total cellular ATP levels under normoxia or hypoxia (n = 5). **(B)** Comparison of mitochondrial ATP and cellular ATP after treatment with vehicle or 20uM UK5099 under normoxia or hypoxia (n = 5). **(C)** Comparison of mitochondrial and cellular ATP after treatment with vehicle or 20uM AZD7545 under normoxia or hypoxia (n = 5). **(D)** Comparison of mitochondrial and cellular ATP after treatment with 10uM PCA under normoxia or hypoxia (n = 6). **(E)** Comparison of mitochondrial and cellular ATP after treatment with vehicle or 20uM SP under normoxia or hypoxia (n = 5). **(F)** Comparison of mitochondrial and cellular ATP after treatment with vehicle or 200nM AA under normoxia or hypoxia (n = 5). Data are presented as Mean ± SEM. *p < 0.05; **p< 0.01; ***p < 0.001 versus vehicle controls (with or without DMSO).
